# Targeting of the β6 gene to suppress degradation of ECM via inactivation of the MAPK pathway in breast adenocarcinoma cells

**DOI:** 10.3892/or.2014.3419

**Published:** 2014-08-20

**Authors:** YUHUA ZHANG, LIJING WEI, JIN YU, GUANG LI, XIURU ZHANG, ANLIU WANG, YANJIAO HE, HONGLI LI, DELING YIN

**Affiliations:** 1 Department of Pathology, Beijing Tiantan Hospital, Capital Medical University, Beijing 100050, P.R. China; 2 Department of Urology, Shandong Provincial Hospital, Jinan, Shandong 250021, P.R. China; 3 Department of Internal Medicine, Department of Pharmacology, James H. Quillen College of Medicine, East Tennessee State University, Johnson City, TN 37614, USA

**Keywords:** breast adenocarcinoma, β6 gene, RNA interference, matrix metalloproteinase, ERK, neoplasm invasion

## Abstract

Integrin ανβ6 has emerged as a potential novel target for anticancer and plays a major role in promoting malignant tumor progression. Recent studies indicate that integrin ανβ6 occurs in many cancers. However, whether and how ανβ6 is regulated by genetic and epigenetic mechanisms in breast cancer remain unknown. In the present study, two different short hairpin RNAs (shRNAs) targeting the β6 gene were designed and constructed into pSUPER, respectively, which were transfected into the MCF-7 human breast adenocarcinoma cell line. The β6-shRNA stably transfected cells were successfully established, and significant lower levels of ανβ6 mRNA and protein expression were confirmed. Furthermore, inhibition of integrin ανβ6 markedly downregulated the expression of matrix metalloproteinase-9 (MMP-9), matrix metalloproteinase-3 (MMP-3) and urokinase plasminogen activator (uPA) in tumor conditioned medium. Furthermore, β6-shRNA-mediated silencing of the ανβ6 gene obviously decreased the expression of ERK1/2. In particular, supression of integrin ανβ6 caused significant downregulation of the degradation of basement membrane type IV collagen secretion via modulation of the plasminogen activation cascade. Our results thus indicate that ανβ6 plays a fundamental role in promoting invasion and growth of breast adenocarcinoma cells. Taken together, this study revealed that targeting of the β6 gene by RNA interference (RNAi) could efficiently downregulate ανβ6 expression and suppress the ERK1/2-dependent extracellular matrix degradation *in vitro*, which is dependent upon inactivation of the mitogen-activated protein kinase (MAPK) pathway. These findings may offer a useful therapeutic approach to block invasion and migration of breast cancer cells.

## Introduction

Breast cancer is currently the most frequently diagnosed cancer and the leading cause of cancer-related death in females worldwide (1). Tumor cell invasion and metastasis are regarded as a multistep process, including the proteolytic degradation of the basement membrane and the extracellular matrix (ECM), altered cell adhesion and the physical movement of tumor cells. Among the many steps involved in tumor invasion and metastasis, excessive degradation of ECM is a crucial step (2).

Matrix metalloproteinases (MMPs) and plasminogen activator (PA) systems, a family of proteinases *in vivo*, are capable of degrading all major components of the ECM (3). These proteinases have been most closely linked with the invasive and metastatic phenotype of cancer cells. Both MMP-9 and MMP-3 have been implicated to play a critical role in breast cancer invasion and metastasis in animal models and human patients (4). Urokinase-type plasminogen activator (uPA) is a 55-kDa serine protease that activates plasminogen to plasmin leading to cell matrix degradation, which is involved in tumor cell adhesion, migration, invasion and intravasation (5).

αvβ6 is one member of the αv integrin subfamily (αvβ1, αvβ3, αvβ5, αvβ6 and αvβ8) defined by the β and αv subunits (6). Integrin αvβ6 is not expressed in normal epithelial cells, but is highly expressed during morphogenetic events, epithelial repair and tumorigenesis (7). Integrin αvβ6 is restrictedly distributed to the epithelium and is typically focally localized at the infiltrating edge of tumor cell islands (8). As a specific epithelial-restricted integrin, αvβ6 has been observed to be upregulated in various invasive epithelial carcinomas of the colon, pancreas, prostate, uterus, lung and breast (9–13). More recent evidence suggests that αvβ6 may be used to identify patients who have a highly significant increased risk of developing metastatic disease and may serve for risk stratification of patients with breast ductal carcinoma *in situ* (DCIS) (14). Therefore, targeting integrin αvβ6 can have unexpected consequences which may represent an opportunity for molecular targeted therapy for aggressive breast carcinoma.

To date, relatively little is known concerning the underlying molecular mechanisms between expression of αvβ6 and degradation of ECM in human breast cancer. Thus, we aimed to explore whether shRNAs targeting αvβ6 can induce gene silencing *in vitro*. In the present study, we investigated the direct effect and transcriptional modulation mechanism of the downregulation of αvβ6 expression on the degradation of ECM.

## Materials and methods

### Reagents

Since the β6 subunit only combines with the αv subunit, the expression of the β6 subunit represents the expression of integrin αvβ6. The anti-αvβ6 mouse anti-human monoclonal antibodies, R6G9 against β6 and 10D5 against αvβ6 (IgG2a), were purchased from Chemicon International (Harrow, UK). The monoclonal antibody against ERK1/2 and phospho-ERK1/2 (Thr202/Tyr204, Thr185/Tyr187) were obtained from Cell Signaling Technology (Boston, MA, USA). N-[(2R)-2-(hydroxamidocarbonylmethyl)-4-methylpentanoyl]-L-tryptophan methylamide (GM6001), amiloride and 1,4-diamino-2,3-dicyano-1,4-bis[2-aminophenylthio] butadiene (U0126) were from Calbiochem (Darmstadt, Germany). Phenol red free (PRF)-DMEM was from Gibco.

### Cell line and cell culture

Human breast adenocarcinoma cell line MCF-7 was kindly provided by Dr Xiaolei Wang, and was obtained from the American Type Culture Collection (Manassas, VA, USA). Cells were maintained in PRF-DMEM supplemented with 10% heat inactivated fetal bovine serum (FBS), 100 U/ml penicillin, 100 μg/ml streptomycin, and 2 mmol/l L-glutamine in a humidified atmosphere with 5% CO_2_ at 37°C. Stably transduced cells were simultaneously cultured in the absence or presence of the indicated concentrations of reagents tested for 72 h in PRF-DMEM containing 10% FBS.

### Design of shRNAs and construction of recombinant vectors

**Figure 1:**
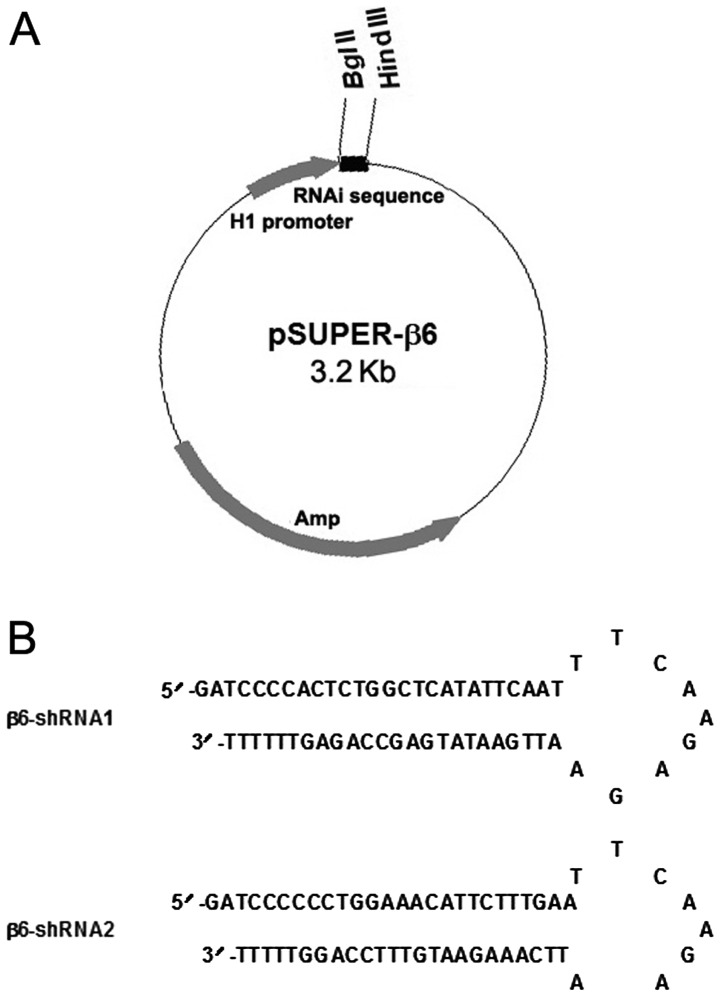
Physical map of the pSUPER-β6 construct. (A) Schematic diagram of siRNA targeting β6 gene expression vector pSUPER-β6. The chemically synthesized stem-loop oligonucleotide was subcloned into pSUPER between *Bgl*II/*Hin*dIII restriction enzyme sites. The expression of β6 siRNA with 19-bp stem and 8-nt loop was driven by Polymerase III-dependent H1 RNA promoter. (B) Sequences of β6-shRNAs.

Because not all small interfering RNA (siRNA) target sequences are equally potent, we designed and selected two different shRNAs targeting different coding regions within the human β6 gene (GenBank accession no. A266609) according to the recommendations (http://www.oligoengine.com and http://sirna.qiagen.com) in our laboratory, which were synthesized by Integrated DNA Technologies (Coralville, IA, USA). BLAST search of genome sequence databases (NCBI Unigene and EST libraries) was performed to ensure that no other human gene was targeted. The double-stranded DNA (dsDNA) was designed as follows: a 19-nucleotide target sequence in both sense and antisense orientations, separated by an 8-nucleotide spacer sequence to form a hairpin dsRNA and flanked at either end by *Bgl*II and *Hin*dIII restriction enzyme sites and the five repeats of T as transcriptional termination signal. The detailed information of these shRNAi used in our study is listed in Table I. The oligonucleotides were then directionally cloned into pSUPER.retro.puro vector at the *Bgl*II and *Hin*dIII sites to generate the β6-shRNA expression vectors (Fig. 1), which were defined as pSUPER-β6shRNA1 and pSUPER-β6shRNA2, respectively. In addition, all constructs were further verified by DNA sequencing.

### Transfection of the siRNA constructs

Stable transfection of the recombinant plasmid pSUPER-β6shRNAs was carried out using Lipofectamine 2000 (Invitrogen, Carlsbad, CA, USA) according to the manufacturer’s protocols. Two hundred and fifty milliliters of DMEM without serum and 4 μg pSUPER-β6shRNAs or control pSUPER per well were pre-incubated for 5 min at room temperature. During the period of preincubation, 250 μl of DMEM without serum was mixed with 10 μl Lipofectamine 2000. The two media were mixed and incubated for 20 min at room temperature for complex formation and then the cells were transfected. Moreover, stable transformants were subsequently selected by growth in medium supplemented with 400 μg/ml geneticin (G418; Gibco) for 3 weeks. After selection with G418, resistant cell clones were then randomly isolated for cell expansion and further analysis.

### RNA extraction and semi-quantitative reverse transcription-PCR analysis

Total cellular RNA was isolated from the untransfected or stably transfected MCF-7 cells using TRIzol (Life Technologies, Carlsbad, CA, USA) according to the manufacturer’s instructions, respectively. A 141-bp fragment of β6 cDNA was amplified with the following primers: forward, 5′-AGGATAGTTCTGTTTCCTGC-3′ and reverse, 5′-ATCATAGGAATATTTGGAGG-3′. As an internal control, amplification of the housekeeping gene glyceraldehyde-3-phosphate dehydrogenase (GAPDH) mRNA was also carried out using the following primers: sense, 5′-GTCGGTGT CAACGGATTTG-3′ and antisense, 5′-ACAAACATGGGGG CATCAG-3′.

### Western blot analysis

Cellular protein extracts from the treated or untreated cells were prepared according to the manufacturer’s protocols (Upstate Biotechnology, Lake Placid, NY, USA). Fifty micrograms of cellular protein per lane was electrophoresed and separated by 4% stacking and 12% resolving SDS-polyacrylamide gel electrophoresis (SDS-PAGE). Separated proteins were electrophoretically transferred and blotted onto a polyvinylidene difluoride filter (PVDF). To avoid unspecific binding, the filters were incubated in 5% skim milk and 0.05% Tween-20 in TBS for 2 h at room temperature. Subsequently, the filters were incubated with mouse monoclonal antibody against human αvβ6 diluted in the same solution (1:1,000) overnight at 4°C and, afterwards, with horseradish peroxidase (HRP)-conjugated goat anti-rabbit IgG secondary antibody diluted at 1:4,000. As control for equivalent protein loading, the filters were simultaneously incubated with mouse anti-GAPDH monoclonal antibody (1:5,000). The level of ERK1/2, phospho-ERK1/2, or uPA expression was also analyzed by western blotting with the monoclonal antibody against ERK1/2, phospho-ERK1/2 or uPA as described above. The protein-antibody complexes were visualized with an enhanced chemiluminescence (ECL) kit (Amersham Pharmacia Biotech) according to the manufacturer’s instructions. The intensity of each band was quantified using an image processing and analysis program.

### Gelatin and casein zymography assays

The expression and activity of MMP-9 and MMP-3 were analyzed by zymography. For assay of MMP-9 (gelatinase B) activity, gelatin zymography and for assay of MMP-3 activity, casein zymography were performed from the samples as described previously (15,16). Briefly, gelatin or casein was added to the 10% acrylamide separating gel at a final concentration of 1 mg/ml for SDS-PAGE, respectively. Tumor conditioned medium (TCM) collected from untreated, pSUPER-controlor pSUPER-β6shRNA-transfected cells under serum-free conditions was mixed with substrate gel sample buffer [10% SDS, 50% glycerol, 25 mM Tris-HCl (pH 6.8) and 0.1% bromophenol blue], and 70 μl was loaded onto the gel without prior boiling. Following electrophoresis, gels were washed twice in 2.5% (v/v) Triton X-100 for 30 min at room temperature to remove the SDS. Gels were then incubated at 37°C overnight in substrate buffer containing 50 mM Tris-HCl and 5 mM CaCl_2_ (pH 8.0). The gels were subsequently stained with 0.15% (w/v) Coomassie brilliant blue R-250 in 50% methanol and 10% glacial acetic acid at room temperature for 20 min, and then destained in the same solution without Coomassie brilliant blue. Gelatin- or casein-degrading enzymes were identified as clear zones in a dark blue background of the stained gel. Zymogram bands were analyzed, and MMP activity was quantified by densitometry (Personal Densitometer SI) and ImageQuant software (both from Molecular Dynamics, Sunnyvale, CA, USA).

### [^3^H]-labeled collagen type IV degradation assay

Collagen type IV degradation assay was performed as previously described (17). In brief, tritium-labeled basement membrane type IV collagen was denatured to form gelatin by heating at 60°C for 30 min. Ninety-six-well plates were coated with 65 μl of [^3^H]-labeled gelatin (15,000 cpm/well) and allowed to dry overnight in a laminar flow hood at room temperature. Plates were then washed 3 times with phosphate-buffered saline (PBS) until free cpm reached the basal level. For cell-mediated collagen degradation assays, untreated, pSUPER-control- or pSUPER-β6shRNA-transfected cells (10^5^ cells/well) were incubated with the gelatin substrates in 300 μl of serum-free DMEM at 37°C for 24 h in the absence or presence of various concentrations of plasminogen. Collagen type IV degradation was determined by subtracting the cpm released from the buffer only wells, and was assessed by measuring the cpm released in 300 μl of medium from triplicate wells for each condition. In studies concerning inhibition of plasminogen activation, uPA and MMP activity, untreated cells and pSUPER-control-treated cells incubated with plasminogen were exposed either to monoclonal antibody against ανβ6 (10D5), uPA inhibitor amiloride (2 mM), MMP inhibitor GM6001 (2 mM), or MEK1/2 inhibitor U0126 for 30 min prior to plating onto the [^3^H]-labeled extracellular matrix. All of the experiments were performed independently and repeated at least three times.

### Statistical analysis

All statistical analyses were performed using SPSS 13.0 software package. The two-sided unpaired Student’s t-test and one-way ANOVA were used to evaluate the statistical significance of differences in two groups and multiple groups, respectively. All data were presented as mean ± standard deviation (SD). P<0.05 was considered to indicate a statistically significant difference.

## Results

### Stable expression of β6shRNAs in the MCF-7 cells

The two shRNAs selected targeting the β6 gene were cloned into the pSUPER.retro vectors (Fig. 1A). The predicted forms and sequences of these shRNAs are shown in Fig. 1B. In addition, the recombinant vectors were validated by restriction enzyme digestion, and the inserted sequences were verified by DNA sequencing. After transfection and selection, the stably transfected cells were named as MCF-7/ανβ6-1 (transfected with pSUPER-β6shRNA1), MCF-7/ανβ6-2 (transfected with pSUPER-β6shRNA2) and MCF-7/CON (transfected with parental vector pSUPER.retro), respectively.

### ανβ6 mRNA expression is efficiently suppressed by pSUPER-β6shRNAs in the MCF-7 cells

**Figure 2:**
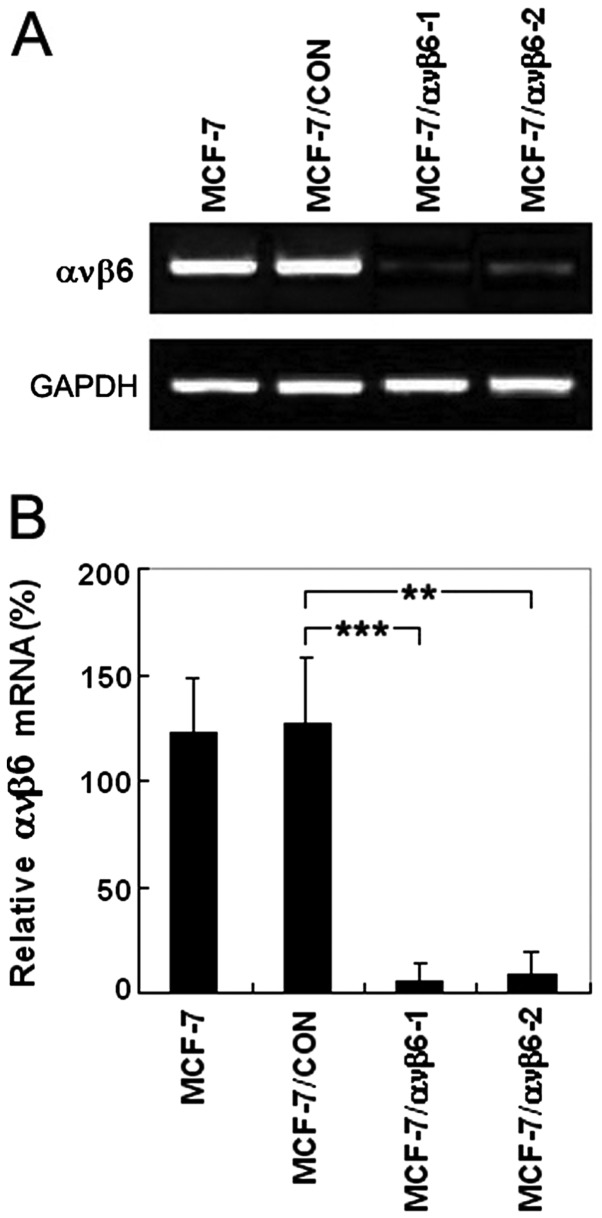
Analysis of shRNA-mediated silencing of ανβ6 mRNA expression in MCF-7 cells. (A) Semi-quantitative RT-PCR showed that the ανβ6 mRNA levels were significantly reduced in the MCF-7/ανβ6-1 and MCF-7/ανβ6-2 cells compared to the level in the MCF-7/CON cells. MCF-7 cells were stably transfected with recombinant plasmid pSUPER-β6shRNA1, pSUPER-β6shRNA2 and parental vector pSUPER.retro, respectively. ανβ6 and GAPDH mRNA levels were then determined by RT-PCR. GAPDH was used as an internal loading control. (B) Relative ανβ6 mRNA levels were normalized against GAPDH expression in the untreated MCF-7 and treated cells (MCF-7/CON, MCF-7/ανβ6-1 and MCF-7/ανβ6-2 cells). Error bars indicate standard deviations. ^*^P<0.05, ^**^P<0.01 and ^***^P<0.001 vs. MCF-7/CON control cells. The ratio of ανβ6 to GAPDH is shown on the y-axis. The data presented are representative of at least three independent experiments.

After β6shRNA stably transfected cells (MCF-7/ανβ6-1 and MCF-7/ανβ6-2) were generated, ανβ6 expression at the mRNA level was investigated by semi-quantitative RT-PCR. In comparison with the control cells, the levels of ανβ6 mRNA were markedly decreased by 95.2 and 91.7% in the MCF-7/ανβ6-1 and MCF-7/ανβ6-2 cells, respectively (Fig. 2A and B). In addition, no effects of RNAi were observed on the expression of GAPDH used as an internal control. These results suggest that β6shRNA can efficiently downregulate ανβ6 mRNA. In summary, shRNA-mediated silencing was markedly pronounced, and no effect could be observed by any other of the controls.

### Knockdown of ανβ6 protein level in the MCF-7 cells by shRNA recombinant plasmids

**Figure 3:**
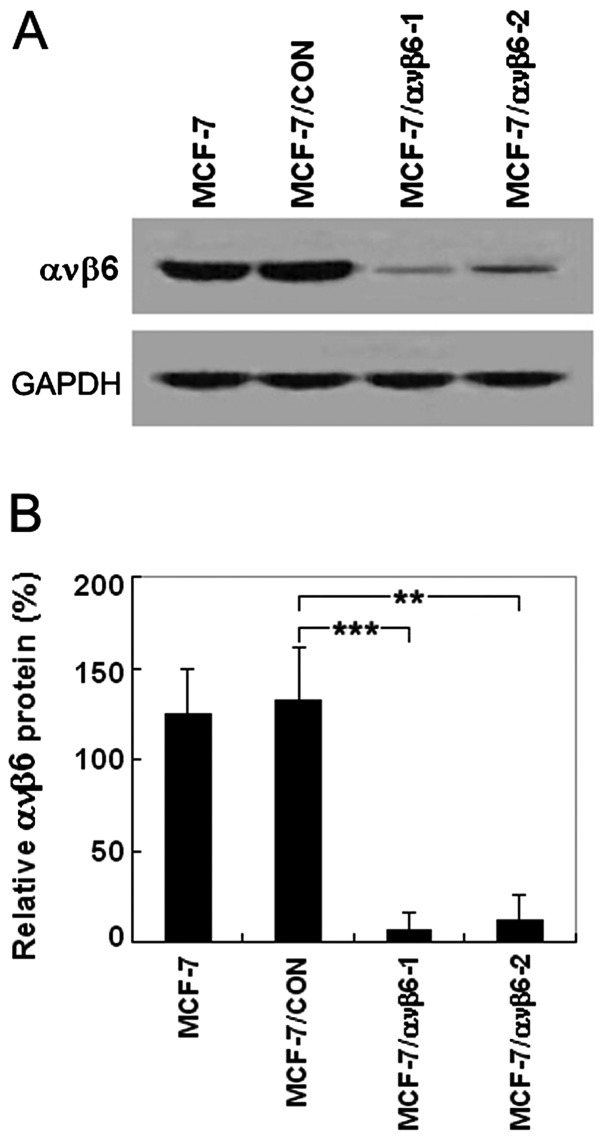
Charaterization of the shRNA-mediated decrease in the ανβ6 protein content in MCF-7 cells. (A) Western blotting showed obviously reduced protein expression of ανβ6 in the untreated MCF-7 and treated cells (MCF-7/CON, MCF-7/ανβ6-1 and MCF-7/ανβ6-2 cells). MCF-7 cells were transfected with β6-shRNA expressing plasmids for 72 h. Cell lysates (50 μg) were then subjected to western blot analysis with the anti-ανβ6 monoclonal antibody 10D5. GAPDH levels were detected as a loading control using the anti-GAPDH monoclonal antibody. (B) Relative ανβ6 protein levels were normalized to GAPDH in the MCF-7/ανβ6-1 and MCF-7/ανβ6-2 cells compared to the level in the MCF-7/CON cells. The error bars indicate standard deviations. Differences in the ανβ6 protein levels are statistically significant: ^*^P<0.05, ^**^P<0.01 and ^***^P<0.001, vs. the control cells. Data are from a single experiment, indicative of at least three independent experiments.

The pSUPER system used in this study was able to markedly suppress the expression of the β6 gene. The shRNA expression plasmids and a control vector were transfected into MCF-7 cells, and ανβ6 protein expression was monitored by western blotting. As shown in Fig. 3A and B, pSUPER-β6shRNA1 and pSUPER-β6shRNA2 significantly decreased the ανβ6 protein level in the MCF-7 cells to 6.9 and 9.7%, respectively, whereas the control vector did not inhibit ανβ6 protein expression. In other words, the ανβ6 protein expression inhibition rates were 93.1 and 90.3% in the MCF-7/ανβ6-1 and MCF-7/ανβ6-2 cells, respectively. However, GAPDH expression was not affected by the same experimental conditions. This change in ανβ6 protein expression was consistent with that of the ανβ6 mRNA level. Therefore, these results indicate that β6shRNA strongly suppresses ανβ6 protein as well as the mRNA level and can be used to target the β6 gene for breast cancer therapy.

### Suppression of integrin ανβ6 expression downregulates ERK1/2 levels

**Figure 4:**
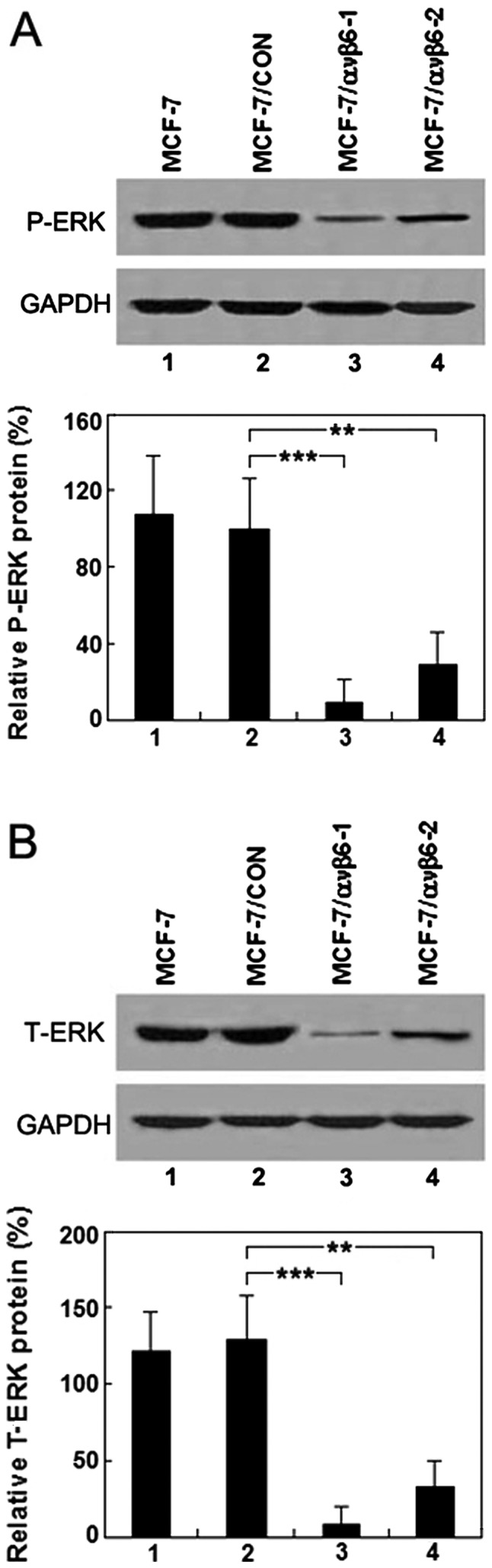
Effect of β6-shRNA on ERK protein expression. (A and B) Immunoblot analysis of total ERK1/2 in the MCF-7 and stably transfected cells (MCF-7/CON, MCF-7/ανβ6-1 and MCF-7/ανβ6-2 cells). Western blot analysis showed that suppression of ανβ6 downregulated the phosphorylation and nonphosphorylation levels of ERK1/2. GAPDH expression was determined as an internal normalization standard for equivalent protein loading. Relative ERK1/2 levels were normalized against GAPDH expression in the MCF-7/ανβ6-1 and MCF-7/ανβ6-2 cells compared to the MCF-7/CON cells. The ratio of ERK1/2 to GAPDH is shown on the y-axis. Error bars show standard deviations. ^*^P<0.05, ^**^P<0.01 and ^***^P<0.001 vs. the control cells. Data are representative of three independent experiments.

To investigate the possible causal link between ανβ6 and ERK1/2, untreated and treated MCF-7 cells for 72 h after stable transfection with pSUPER-β6shRNAs or pSUPER-control were harvested. The levels of ERK1/2 and phospho-ERK1/2 were assessed by western blot analysis. The inhibitory effects of the downregulation of ανβ6 expression on the phosphorylation and nonphosphorylation levels of ERK1/2 in the MCF-7/ανβ6-1 and MCF-7/ανβ6-2 cells are shown in Fig. 4A and B. These findings are in agreement with the change in ανβ6 mRNA and protein levels and further support our hypothesis that the suppression of ανβ6 expression by β6shRNAs leads to the inactivation of the MAP kinase pathway.

### Inhibition of integrin ανβ6 suppresses the secretion of pro-MMP-9, pro-MMP-3 and uPA in tumor conditioned medium from the human breast cancer MCF-7 cell line

**Figure 5:**
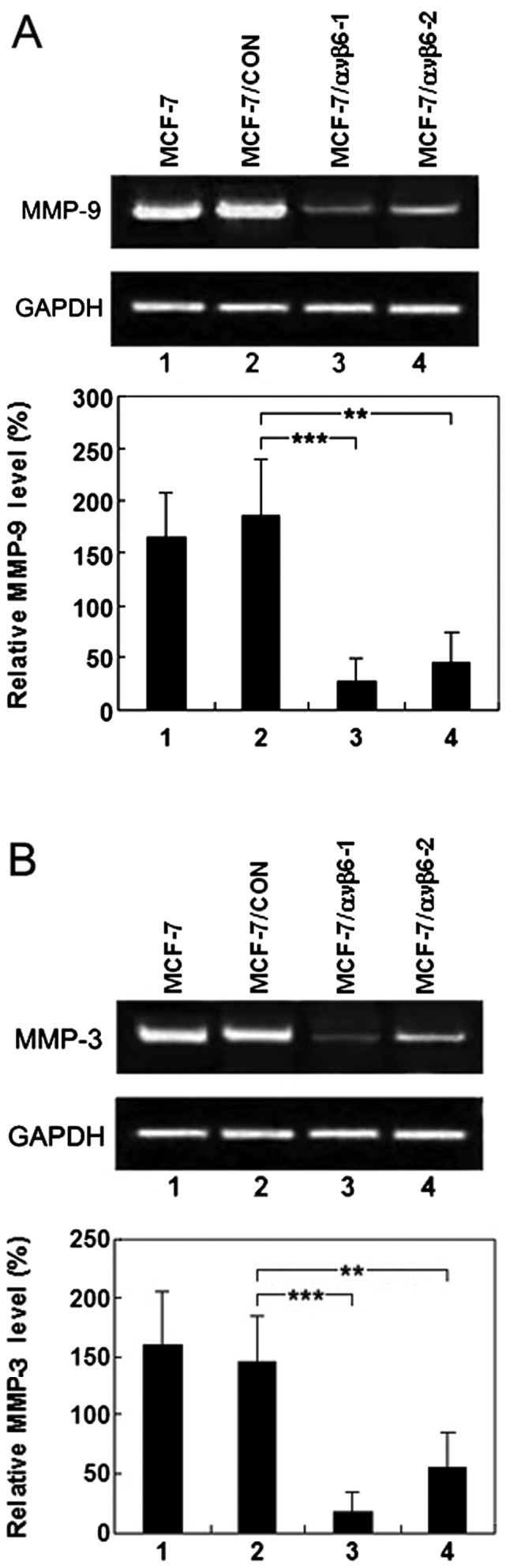
Effects of β6-shRNA on MMP expression. (A and B) Knockdown of integrin ανβ6 suppresses the secretion of pro-MMP-9 and pro-MMP-3 in tumor conditioned medium from MCF-7 cells. MCF-7 cells were stably transfected with pSUPER-β6shRNA1, pSUPER-β6shRNA2 or pSUPER-control for 72 h, were harvested and TCM was prepared. Gelatin and casein zymography assays were used to assess the expression and activity of MMP-9 and MMP-3. GAPDH was considered as an internal normalization standard. MMP-9 and MMP-3 production in MCF-7/ανβ6-1 and MCF-7/ανβ6-2 cells was markedly decreased when compared to production in the MCF-7/CON control cells. Relative MMP levels were normalized against GAPDH expression in the untreated MCF-7 and treated cells (MCF-7/CON, MCF-7/ανβ6-1 and MCF-7/ανβ6-2 cells). The ratio of MMPs to GAPDH is shown on the y-axis. Results are shown as mean values ± SEM of three experiments performed in triplicate. ^*^P<0.05, ^**^P<0.01 and ^***^P<0.001, vs. the control cells.

**Figure 6:**
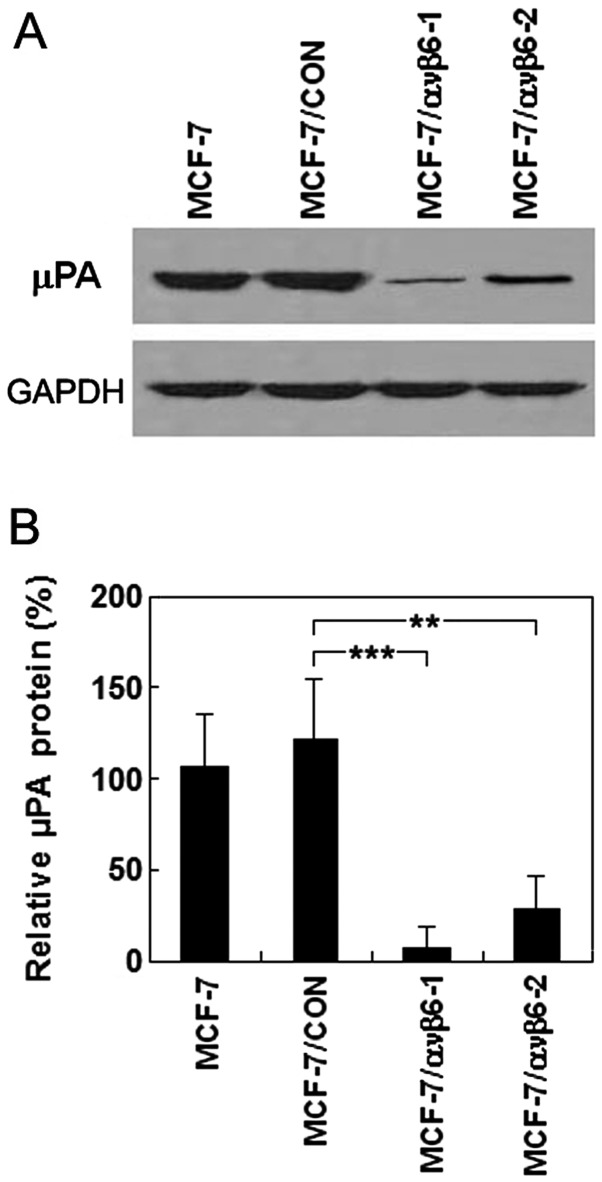
Effects of β6-shRNA on uPA expression. (A) Silencing of integrin ανβ6 inhibited the secretion of uPA in tumor conditioned medium from MCF-7 cells. MCF-7 cells were treated with pSUPER-β6shRNA1, pSUPER-β6shRNA2 or pSUPER-control for 72 h and TCM was prepared. A protein band of ~55 kDa was monitored by western blotting. GAPDH was also determined as a control for equivalent protein loading. The levels of uPA in MCF-7/ανβ6-1 and MCF-7/ανβ6-2 cells were significantly lower than that of the MCF-7/CON control cells. (B) Relative uPA expression was normalized against GAPDH level in the untreated MCF-7 and treated cells (MCF-7/CON, MCF-7/ανβ6-1 and MCF-7/ανβ6-2 cells). Error bars show standard deviations. ^*^P<0.05, ^**^P<0.01 and ^***^P<0.001 vs. the control cells. Data are indicative of three independent experiments.

The effects of reduced ανβ6 on MMP-9, MMP-3 and uPA expression *in vitro* were evaluated by gelatin zymography, casein zymography and western blot analysis, respectively. Untreated MCF-7 cells and cells after stable transfection with pSUPER-β6shRNA1, pSUPER-β6shRNA2 or pSUPER-control for 72 h were harvested and TCM was prepared. As shown in Fig. 5A and B, compared with the control cells, MMP-9 and MMP-3 production was decreased by 90.7 and 93.8% in the MCF-7/ανβ6-1 cells, respectively. Next, we aimed to ascertain whether a similar trend would be observed in the MCF-7/ανβ6-2 cells, stably transfected with pSUPER-β6shRNA2. MMP-9 and MMP-3 production was reduced by 70.4 and 75.6%, respectively (Fig. 5A and B). Furthermore, western blot analysis demonstrated that relative uPA protein levels were 7.1±0.6 and 28.3±1.2% in the MCF-7/ανβ6-1 and MCF-7/ανβ6-2 cells, respectively, significantly lower than that of the control cells (121.4±3.5%; P<0.05) (Fig. 6A and B). In other words, the uPA protein expression was decreased by 94.2 and 76.7% in the MCF-7/ανβ6-1 (transfected with pSUPER-β6shRNA1) and MCF-7/ανβ6-2 cells (transfected with pSUPER-β6shRNA2), respectively, compared with that of the MCF-7/CON cells (transfected with parental vector pSUPER.retro). No effects of RNAi were observed in regards to the expression of GAPDH, which was used as an internal control. Therefore, these results suggest that inhibition of integrin ανβ6 by RNAi could efficiently suppress the secretion of pro-MMP-9, pro-MMP-3 and uPA in the human breast cancer MCF-7 cell line.

### Effect of ανβ6 gene expression silencing by RNAi on degradation of [^3^H]-labeled collagen type IV

**Figure 7:**
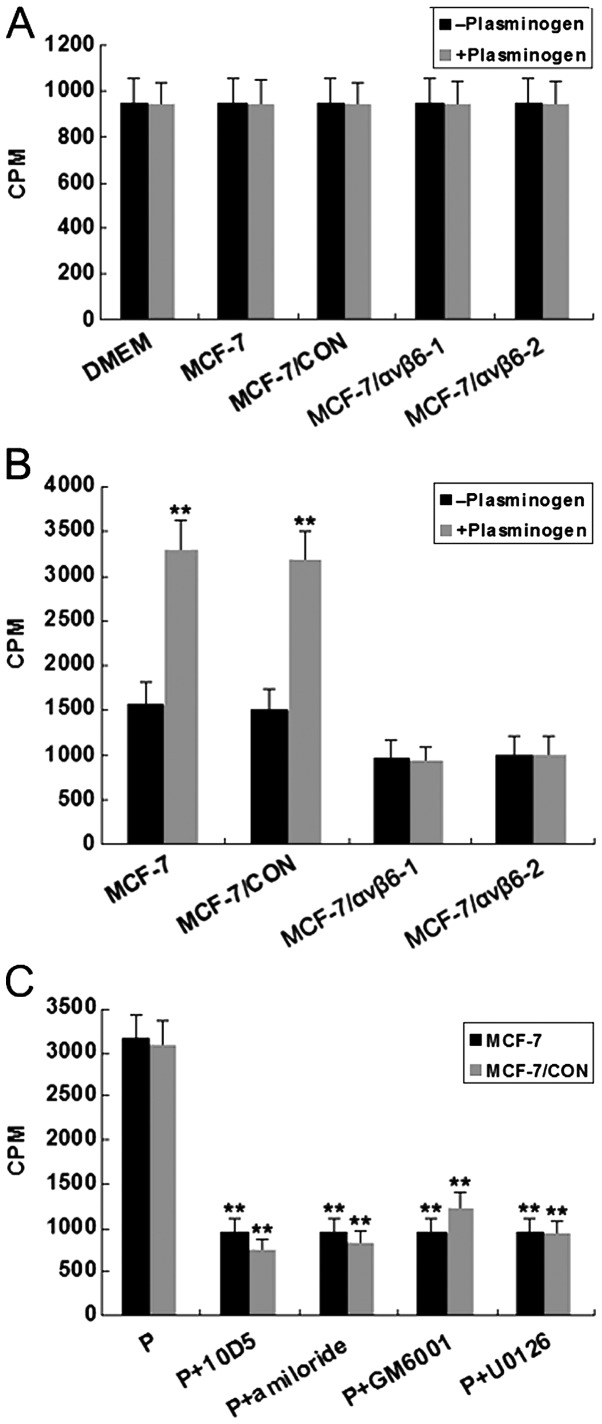
Effect of ανβ6 gene expression silencing by RNAi on the degradation of [^3^H]-labeled collagen type IV. Collagen type IV degradation was measured by the release of tritium into the fluid phase, and triplicate wells were used for each experimental condition. (A) Untreated MCF-7 and treated cells (MCF-7/CON, MCF-7/ανβ6-1 and MCF-7/ανβ6-2 cells) were harvested and incubated for 24 h in the absence or presence of plasminogen (8 μg/ml) in 24-well plates coated with [^3^H]-labeled heat-denatured collagen type IV. Background cpm (spontaneous release of tritium in the presence of DMEM) is shown on the left. ^**^P<0.01, vs. the corresponding control cells in the absence of plasminogen. (B) Untreated MCF-7 and treated cells (MCF-7/CON, MCF-7/ανβ6-1 and MCF-7/ανβ6-2 cells) were incubated for 24 h in the absence or presence of plasminogen (8 μg/ml) in 24-well plates coated with [^3^H]-labeled, heat-denatured collagen type IV. ^**^P<0.01, vs. the corresponding control cells in the absence of plasminogen. (C) Untreated cells and pSUPER-control treated cells incubated with plasminogen (P, 8 μg/ml) were exposed either to a monoclonal antibody against ανβ6 (10D5), uPA inhibitor amiloride (2 mM), MMP inhibitor GM6001 (2 mM), or MEK1/2 inhibitor U0126 for the duration of the experiment. Results are shown as mean values ± SEM of three different experiments performed in triplicate. ^**^P<0.01, vs. the corresponding control cells in the absence of plasminogen.

To determine whether inhibition of integrin ανβ6 by RNAi suppresses extracellular matrix degradation, plasminogen-dependent [^3^H]-labeled collagen type IV degradation assay was performed. Collagen type IV, the major structural component of the basement membrane, was used as the substrate for both collagenase MMP-9 and MMP-3. Degradation of the basement membrane was measured by the release of tritium from [^3^H]-labeled, heat-denatured radiolabeled type IV collagen. Exposure of the gelatin substrate to serum-free nonconditioned culture medium DMEM for 24 h resulted in spontaneous, non-proteinase-mediated release of tritium into the fluid phase background cpm, the counts per minute measured (Fig. 7A). Exposure of the collagen substrate to TCM obtained from the untreated cells, pSUPER-β6shRNA- and pSUPER-control transfected cells did not result in tritium release in either the presence or absence of 8 μg/ml plasminogen above background levels (Fig. 7A), indicating that the released collagenases in the culture supernatants were neither active nor activatable by plasminogen in the absence of cells. In contrast, exposure of collagen to untreated and pSUPER-control-treated human breast cancer MCF-7 cells in the presence of exogenous plasminogen significantly increased the basal level of collagen type IV degradation, compared to the corresponding control cells in the absence of plasminogen.

Not unexpectedly, there was no such effect in the pSUPER-β6shRNA-transfected cells (Fig. 7B). Furthermore, as shown in Fig. 7C, the increased and more extensive collagen degradation monitored in the untreated and pSUPER-control-treated cells, was abolished by the addition of either anti-αvβ6 antibody 10D5, MMP inhibitor GM6001, uPA inhibitor amiloride or MEK1/2 inhibitor U0126. These results strongly suggest that silencing of ανβ6 gene expression by RNAi could effectively suppress mitogen-activated protein kinase (MAPK)-dependent degradation of the extracellular matrix, and also demonstrated that MMPs and uPA-mediated plasminogen dependent-proteolysis are essential to the degradation of the basement membrane.

## Discussion

Degradation of extracellular matrix components and basement membranes is crucial for invasion and metastasis of cancer cells. During this process, MMPs appear to be primarily responsible for much of the ECM degradation (18). Recently, integrin ανβ6 has emerged as a novel potential target for anticancer therapy and plays a major role in promoting malignant tumor progression. In addition, it has previously been reported that integrin αvβ6 is restrictedly distributed to the epithelium and is typically focally localized at the infiltrating edge of tumor cell islands.

In the present study, we further investigated the effect and regulatory mechanism of ανβ6 gene knockdown by RNAi on the degradation of the extracellular matrix in MCF-7 breast cancer cells. RNAi is a novel and powerful tool for specific inhibition of a targeted gene by introducing double-stranded RNA into cells leading to sequence-specific destruction (19–21). RNAi is a sequence-specific, post-transcriptional gene silencing process in many organisms, which can be triggered by dsRNA and is cleaved into 21- to 23-nucleotide RNA fragments, known as siRNAs, by the ribonuclease III (RNase III)-like enzyme, Dicer. Subsequently, these siRNAs are incorporated into a protein complex, which is also called RNA-induced silencing complex (RISC). This protein complex is able to degrade homologous mRNA and then to inhibit the targeting gene at the post-transcriptional level (22,23). RNAi constitutes a promising source for new therapeutic approaches, including cancer gene therapy. For years, many research groups have focused on effective tools to specifically downregulate gene expression, such as antisense oligonucleotide strategy. However, its success has been limited, due to the lack of specificity and potency. RNAi-induced knockdown of target gene expression is an attractive approach for gene therapy since multiple targets may be manipulated simultaneously (24–26). Therefore, we explored whether shRNAs targeting αvβ6 can induce gene silencing *in vitro*.

The present results demonstrated that siRNA can efficiently suppress ανβ6 expression with high specificity at the mRNA and protein levels. Because of the pSUPER vector containing the H1 RNA polymerase III promoter upstream of the inserted DNA sequence, the shRNAs can be effectively expressed after being transfected into tumor cells (27). In our study, by using a new retroviral pSUPER.retro vector system, we successfully generated permanent cell lines that constitutively express specific siRNA. First, two shRNAs targeting the ανβ6 gene were selected and cloned into the expression vector pSUPER. We successfully established stably transfected cells: MCF-7/ανβ6-1, MCF-7/ανβ6-2 and MCF-7/CON. In addition, our data showed that both shRNAs against αvβ6 expressed by the recombinant plasmids were markedly effective in suppressing αvβ6 mRNA and protein in MCF-7 cells. RT-PCR detection revealed that pSUPER-β6shRNA1 and pSUPER-β6shRNA2 decreased ανβ6 mRNA by 95.2 and 91.7%, respectively. ανβ6 protein expression was reduced by 93.1 and 90.3% in the MCF-7/ανβ6-1 and MCF-7/ανβ6-2 cells compared to the MCF-7/CON cells, respectively, by western blot analysis. Relative ανβ6 mRNA and protein levels were significantly lower than these levels in the untreated control cells. In this experiment, both pSUPER-β6shRNA constructs showed a pronounced ανβ6 gene silencing activity in the MCF-7 cells at the mRNA level as well as the protein level and were similar to each other. These data thus indicate that ανβ6 could specifically serve as a biomarker and a potential and attractive therapeutic target for directed cancer genetic therapy.

Integrin ανβ6 in cancer cells is of particular interest for the fact that it is not expressed in normal epithelium, but is highly expressed during tumorigenesis, leading to enhanced tumor cell migration and invasion (28). Recently, it has been reported that the upregulation of the integrin β6 gene could be involved in oxytocin-induced cell growth in human breast tumor-derived endothelial cells (29). A more recent study also showed that ανβ6-positive patients have a markedly high risk of progression to invasive breast cancer (14). To date, it remains unclear whether and how ανβ6 is involved in regulation of breast cancer cell migration and metastasis, or whether there is any relationship between ανβ6 expression and ECM degradation in human breast cancer cells. In the present study, the effect and possible regulatory mechanism of the downregulation of ανβ6 expression on matrix degrading enzyme secretion in breast cancer cells were investigated. Degradation of the extracellular matrix and components of the basement membrane by proteases facilitates the detachment of tumor cells, their crossing of tissue boundaries, and invasion into adjacent tissue compartments. MMPs and the serine protease uPA, the class of proteolytic enzymes, are thought to play a central role in this process, because of their ability to degrade many ECM components (30). In the present study, MMP-9, MMP-3 and uPA protein expression levels were decreased by 93.1, 90.3 and 94.2% in the MCF-7/ανβ6-1 cells, and 70.4, 75.6 and 76.7% in the MCF-7/ανβ6-2 cells compared to the MCF-7/CON cells, respectively. To the best of our knowledge, we showed for the first time that suppression of integrin ανβ6 in MCF-7 human breast carcinoma cells dramatically reduced pro-MMP-9, pro-MMP-3 and uPA secretion in tumor conditioned medium. These findings are in agreement with the observation by Gu *et al* that inhibition of β6 expression, MEK inhibition, or deletion of the β6-ERK2 binding site suppressed MMP-9 secretion in human colon cancer cell lines WiDr and HT29 (31). Our results thus indicate a potential regulatory mechanism whereby integrin ανβ6 contributes to breast cancer invasion by enhancing matrix degrading enzyme secretion and activity.

In addition, to assess whether downregulation of pro-MMP-9, pro-MMP-2, uPA expression in serum-free tumor conditioned medium affects the degradation of the extracellular matrix, radiolabeled type IV collagen degradation assay was performed. Loss of basement membrane type IV collagen has previously been shown to be associated with breast cancer tumorigenesis, and the absence of type IV collagen was found to be involved in the overexpression of MMPs. uPA has been identified as the critical trigger for active plasmin generation resulting in an overall increase in catalytic efficiency (32). Moreover, it has been recently reported that activation of pro-MMPs by plasminogen occurs through uPA-mediated generation of plasmin (33). In the present study, the plasminogen-dependent ECM degradation in the untreated and pSUPER-control cells was completely abrogated by the uPA inhibitor, anti-MMPs or anti-ανβ6, suggesting that the inhibitory effects of downregulated ανβ6 expression was via the plasminogen activation cascade. Therefore, our results also demonstrated that inhibition of ανβ6 expression in breast cancer MCF-7 cells suppresses the plasminogen-dependent degradation of the extracellular matrix.

The MAPK signaling pathway, a family of protein-serine/threonine kinases, plays a fundamental role in regulating cellular proliferation, differentiation, migration and apoptosis (34). MAPKs are involved in the regulation of MMP expression associated with invasion of malignant tumor cells (35). It is now becoming clear that activation of epidermal growth factor receptor (EGFR) and its subsequent regulation of extracellular signal-regulated kinases (ERKs) for cell survival is dependent on integrin-mediated, ligand-independent signal transduction involving MAPK cascades (36). Moreover, it is important that integrin ligation induces activation of ERKs when the concentration of growth factors available to the cell is limited. Either deletion of the ERK2 binding site on the β6 cytoplasmic domain or downregulation of β6 expression inhibits tumor growth and may be due to an association between ERK and the β6 subunit (37). This is also supported by the finding that integrin-mediated MMP-9 secretion is dependent upon direct binding between the β6 integrin subunit and ERK2 (31). The data in this study show that downregulation of integrin ανβ6 expression by pSUPER-β6shRNAs significantly reduced the levels of phosphorylated and unphosphorylated ERK1/2. Furthermore, plasminogen-dependent ECM degradation of untreated and pSUPER-control treated cells was almost completely abolished by the specific MEK1/2 inhibitor U0126, indicating that suppression of ECM degradation was induced by inhibition of integrin ανβ6 expression dependent on MAPK activity.

In summary, our findings thus demonstrated that RNA technology-based approach for suppression of the ανβ6 gene *in vitro* can efficiently downregulate ανβ6 expression in breast cancer, and in the future may offer a useful therapeutic strategy to block invasion and migration for the treatment of breast cancer. In addition, our study revealed that inhibition of the ανβ6 gene markedly decreased the activity of MMP-9, MMP-3, uPA and the ERK1/2-dependent degradation of ECM. These data therefore provide new insight into the regulatory mechanism of integrin ανβ6 in ECM degradation, which might be involved in inactivation of the MAP kinase pathway in the progression of human breast cancer.

## Figures and Tables

**Table I:** Oligonucleotide sequences of the shRNAi constructs.

β6-shRNA1	
Sense	5′-gatccccACTCTGGCTCATATTCAATTCAAGAGAATTGAATATGAGCCAGAGTtttttgaa-3′
Antisense	5′-agctttcaaaaaACTCTGGCTCATATTCAATTCAAGAGAATTGAATATGAGCCAGAGTggg-3′
β6-shRNA2	
Sense	5′-gatccccCCTGGAAACATTCTTTGAATCAAGAGATTCAAAGAATGTTTCCAGGtttttgaa-3′
Antisense	5′-agctttcaaaaaCCTGGAAACATTCTTTGAATCAAGAGATTCAAAGAATGTTTCCAGGggg-3′
